# Lifestyle behavior of physiotherapy students from Ecuador upon admission to higher education: a cross-sectional study

**DOI:** 10.3389/fspor.2024.1352144

**Published:** 2024-04-05

**Authors:** Héctor Gutiérrez-Espinoza, María Cassola-Cajiao, Emilia Garzón-Ulloa, Daniela Celi-Lalama, Carlos Bastidas-Caldes, Felipe Araya-Quintanilla, Carlos Cristi-Montero, José Francisco López-Gil

**Affiliations:** ^1^ Escuela de Fisioterapia, Universidad de las Americas, Quito, Ecuador; ^2^ One Health Research Group, Universidad de las Americas, Quito, Ecuador; ^3^ Escuela de Kinesiología, Facultad de Odontología y Ciencias de la Rehabilitación, Universidad San Sebastián, Santiago, Chile; ^4^ IRyS Group, Physical Education School, Pontificia Universidad Católica de Valparaíso, Valparaíso, Chile; ^5^ Department of Communication and Education, Loyola University, Andalusia, Seville, Spain

**Keywords:** 24-hour movement guidelines, lifestyle behavior, anthropometrics, body composition, university students, cross-sectional study

## Abstract

**Introduction:**

Admission to university has been identified as a period involving the adoption of unhealthy lifestyle behaviors. However, few studies have addressed the extent of this experience among Latin American university students. The aim of this study was twofold: first, to describe anthropometric variables, body composition, physical activity, sedentary behavior, sleep duration and quality, diet, and alcohol consumption in first-year students entering physiotherapy school at the *Universidad de las Americas* in Quito, Ecuador; second, to test differences in these variables between sexes.

**Methods:**

A total of 116 students were recruited. Sociodemographic variables, anthropometric indices, body composition, physical activity, sedentary behavior, sleep quality and duration, adherence to 24-hour movement guidelines, physical fitness (i.e., handgrip strength), diet, alcohol consumption, and smoking habits were evaluated.

**Results:**

A total of 50 male (43.1%) and 66 female (56.9%) students were assessed. Overall, the adherence to the 24-hour movement guidelines of the students upon admission to university was 8.6%. Conversely, 86 students (74.1%) did not meet any of the recommendations. Upon admission to university, only 8.6% of the students (female 2.6%; male 6%) met the overall 24-hour movement guidelines. Additionally, 82 students (70.7%) needed changes in diet quality, 81 students (69.8%) had significant sleep disturbances, and 22 students (18.9%) had harmful alcohol consumption. A greater proportion of males met all three 24-h movement recommendations (*p* = 0.025) than females did. In addition, females reported a greater percentage of occasional smokers (*p* = 0.025) and a greater prevalence of obesity (*p* < 0.001), a lower level of physical activity (*p* < 0.001), and a greater percentage of sleep disturbance (*p* < 0.001). Conversely, males reported greater waist circumference (*p* = 0.005), weight (*p* < 0.001), handgrip strength (*p* < 0.001), and a greater percentage of harmful alcohol consumption (*p* < 0.001).

**Discussion:**

Our study showed that upon admission to university, overall adherence to the 24-hour movement guidelines is low among university students. Additionally, a high percentage of students reported unhealthy lifestyle behaviors, with differences according to sex. Our findings could lead to the use of specific sex-specific strategies for the prevention and promotion of movement and lifestyle behaviors during this critical period.

## 1 Introduction

University represents a transition period for young adults that involves behavioral adaptation in a new environment, with new relationships and changing support systems (1). This period is characterized by important lifestyle changes (2). Indeed, when analyzing patterns of lifestyle change over the lifespan, a critical time for unhealthy lifestyle behaviors appears to be in late adolescence and early adulthood (3–5). In this sense, admission to university is a critical period because it is associated with unhealthy lifestyle behaviors, during which students experience changes in eating behaviors, physical activity levels, sleep patterns, and increased alcohol consumption (5–7).

Consequently, several systematic reviews have reported that admission to higher education is usually accompanied by changes in eating behavior, body composition, body weight, and level of physical activity (8–11). There has been considerable interest in recent decades in the “Freshman 15” phenomenon, the popular belief that United States students gain 15 lb (6.8 kg) of weight in their first year of university (8). Currently, studies performed in numerous countries have offered evidence to support this finding (9); however, the magnitude of the effect has decreased to 3.38 kg, without statistically significant differences according to sex (9). In addition to weight gain, a meta-analysis that included 19 studies from several countries showed that there was an increase in fat intake of 1.17% during the first years of university (10).

Some studies have reported that admission to higher education is usually accompanied by changes in diet quality or eating behaviors (12–15). For instance, one study showed a decrease in the consumption of fruits, vegetables and dairy products (12). Similarly, another study showed a decrease in the consumption of fruit and dairy products but not meat, grains, vegetables, coffee/tea or soft drinks (13). Finally, two studies showed a small decrease in meal and snack consumption and a decrease in total energy intake (14, 15). A meta-analysis of physical activity showed that leaving secondary school was associated with a decrease of 7.04 min/day in moderate-to-vigorous physical activity over an average follow-up of 0.8 years (11). Additionally, a subgroup analysis showed that this decrease was greater in males than in females (11). Studies emphasize that the amount of time spent in front of computer screens and televisions is increasing, which, combined with a lack of physical activity, has a negative impact on the quality of life of university students (16, 17). According to these findings, a global survey performed in 23 countries with different income levels showed that between 21.9% and 80.6% of university students were considered as physically inactive (18).

At present, there is extensive documentation highlighting the positive health benefits associated with high levels of physical activity, limited sedentary time, and an optimal duration of sleep (19). It is crucial to recognize that these three aspects of movement behavior are interconnected as they occur throughout the entire 24-hour day, and thus, they should be considered collectively (19, 20). Acknowledging the significance of optimal time allocation for overall health, the Canadian 24-hour movement guidelines for adults aged 18–64 recommend 150 min per week of moderate-to-vigorous physical activity, no more than eight hours of daily sedentary time, and seven to nine hours of nightly sleep (19). Adhering to all three guidelines has been linked to positive outcomes in cardiometabolic health, adiposity, physical fitness, mental health, and the risk of mortality among adult and older adult populations (21).

On the other hand, according to the World Health Organization, a healthy lifestyle involves regular physical exercise, abstention from smoking, limiting alcohol consumption, and eating healthy foods to prevent excess body weight gain (22). In this sense, several studies suggest that attending university is not a factor promoting a healthy lifestyle (23–25). Therefore, university students are a social group that is at risk from a lifestyle behavior point of view since they often do not follow healthy eating patterns, exhibit low levels of physical activity, and exhibit high levels of alcohol consumption, with differences according to sex (5–7, 26). Considering these findings, new responsibilities should accompany higher education in the prevention and promotion of healthy lifestyle behaviors.

To our knowledge, only a few published studies have analyzed adherence to 24-hour movement guidelines and anthropometrics, body composition, and lifestyle behavior upon admission to university among students in Latin American countries, and especially none have been published in Ecuador. Given the scarcity of published data about these variables, it seems necessary to obtain information from Ecuadorian university students. Therefore, the aim of this study was twofold: first, to describe anthropometric variables, body composition, sleep duration and quality, sedentary behavior, physical activity, diet, and alcohol consumption in first-year students entering physiotherapy school at the *Universidad de las Americas* in Quito, Ecuador; second, to test differences in these variables between sexes.

## 2 Materials and methods

### 2.1 Design/setting

The current study was conducted following the Strengthening the Reporting of Observational Studies in Epidemiology (STROBE) guidelines for cross-sectional studies (27). The study was approved by the Ethics Committee for Research on Human Beings of Universidad de las Americas of Ecuador (ID: 2022-OBS-008) and received funding from the Universidad de las Americas under sponsor code FIS.HGE.23.01. In the second semester of 2022 and the first semester of 2023, a total of 116 university students were recruited. All participants signed an informed consent form approved by the ethics committee.

### 2.2 Participants

We included all students older than 18 years who entered physiotherapy school at the Universidad de las Americas in the city of Quito, Ecuador during two academic semesters. Conversely, the following were excluded: (i) had any uncontrolled comorbidities, such as hypertension, diabetes mellitus, or hypercholesterolemia; or (ii) had physical impairments associated with neurological diseases or mental illness.

### 2.3 Outcome measures

Prior to the start of the study, two researchers with postgraduate in Kinanthropometry received 2-week training was carried out to standardize the measurements.

#### 2.3.1 Sociodemographic and medical condition variables

Age, sex, socioeconomic status (high, middle, or low), and work occupation (full-time, part-time, or not working) were assessed for each participant. Comorbidity data, including hypertension, diabetes mellitus, and hypercholesterolemia, were also assessed.

#### 2.3.2 Anthropometric variables

While participants stood still on a flat surface, their waist circumference was measured using a no stretchable tape, positioned midway between the costal margins and the iliac crest in the horizontal plane. Height was measured barefoot and upright with a Seca 213 stadiometer, ensuring the sagittal midline touched the backboard. Body weight, measured with a Tanita HD-351, was taken with participants barefoot and in light clothing. These measurement procedures align with the recommendations of the International Society for the Advancement of Kinanthropometry. Finally, body mass index (BMI) was computed as the weight in kilograms divided by the square of height in meters, and the BMI values were used to determine nutritional status following the World Health Organization standards (28).

#### 2.3.3 Body composition

The noninvasive assessment of body composition utilized the Seca Medical Body Composition Analyzer 525, employing phase-sensitive, multifrequency technology, and operated with Seca Analytics 115 software (Seca GmbH & Co., Hamburg, Germany). The Seca mBCA 525 device employed four pairs of standard surface electrodes, attached twice to each hand and foot, all connected to a computer analyzer. Measurements were conducted under standardized conditions, with participants lying in a supine position on a nonconductive surface. The specified body position involved lower extremities at a 45° angle to each other and upper extremities at a 30° angle relative to the trunk, following 10 min of rest (29). Key variables, including fat mass index, lean mass index, total body water, and phase angle, were recorded during these measurements. Although, it is not the gold standard method to assess body composition, it shows a high test-retest reliability (30).

#### 2.3.4 Physical activity and sedentary behavior

The Global Physical Activity Questionnaire (GPAQ) served as the tool for assessing the participants' physical activity levels (31). This validated 16-item scale gauges moderate- and vigorous-intensity physical activity across three distinct domains: work, recreation, and active transportation, over the course of a typical week (31). The GPAQ has been established as a reliable and valid instrument specifically designed for measuring physical activity in university students (32).

#### 2.3.5 Sleep duration and quality

The assessment of sleep duration and quality utilized the Pittsburgh Sleep Quality Index (PSQI) questionnaire (33). This self-rated questionnaire consists of 19 items and evaluates sleep quality over a one-month period. The total score, ranging from 0 to 21, indicates sleep quality, with higher scores suggesting poorer sleep quality. A total score exceeding five points indicates significant sleep disturbance (33). The Spanish version of the PSQI is recognized as a valid and reliable instrument for measuring sleep quality (34).

#### 2.3.6 Adherence to 24-hour movement guidelines

In adherence to the Canadian 24-hour movement guidelines for adults, participants were obligated to fulfill the following conditions: (a) engage in at least 150 min per week of moderate-to-vigorous physical activity, incorporating muscle-strengthening activities at least twice a week; (b) limit sedentary time to no more than 8 h daily and recreational screen time to no more than 3 h daily; and (c) maintain a nightly sleep duration of 7 to 9 h (19). Based on data obtained from personal assessments and two self-report questionnaires (31, 33), participants meeting all three criteria were categorized as compliant with the recommendations.

#### 2.3.7 Physical fitness

Physical fitness, specifically handgrip strength, was evaluated using a JamarTM dynamometer (Model FS360). Participants assumed a stance with their feet approximately shoulder-width apart, utilizing a wall behind them to enhance stability, prevent trunk rotation, and maintain arm position. The measurements were conducted with the arm and wrist in a neutral position and the elbow flexed at 90° (35). Both the dominant and nondominant sides were assessed, with verbal encouragement provided to ensure maximal contraction lasting five seconds. A rest time of one minute between each contraction allowed for sufficient recovery, and the result was the average value obtained from three separate attempts (36).

#### 2.3.8 Diet

Diet quality was assessed with the Spanish version of the Healthy Eating Index (HEI) (37). Participants' diets were classified according to the total score and divided into three categories: >80 points, “healthy”; 50–80 points, “needs changes”; and <50 points, “unhealthy.” This is a valid and reliable instrument for measuring dietary quality (38).

#### 2.3.9 Alcohol consumption

The evaluation of alcohol consumption was conducted using the Alcohol Use Disorders Identification Test (AUDIT) (39). Comprising 10 items, this questionnaire is specifically designed to assess hazardous alcohol intake. Scores on the AUDIT range from 0 to 40, with higher scores indicative of an elevated risk of problematic alcohol consumption. A total score exceeding eight points is considered a cutoff point indicating harmful alcohol consumption (40). The Ecuadorian adaptation of the Spanish version of the AUDIT has demonstrated good internal consistency and reliability (41).

#### 2.3.10 Smoking habits

Smoking habits were evaluated according to the C4 smoking classification questionnaire (dependent, heavy, moderate-risk, or occasional smoker) (42). This instrument has not been evaluated for validity and reliability in university students.

### 2.4 Statistical analysis

Descriptive statistics were employed to characterize the sociodemographic, medical condition, anthropometric, body composition, and lifestyle variables among the participants. Continuous variables are reported as the mean and standard deviation (SD), while categorical variables are expressed as the number and percentage (%). The choice of statistical tests for data analysis involved the utilization of both the Shapiro–Wilk test and graphical procedures such as the normal probability plot to assess normality. In comparing data between males and females, Student's *t*-test was applied for quantitative variables, and the chi-square test was employed for categorical variables. All the statistical analyses were performed using Stata 16.0 (Stata, College Station, TX, USA).

## 3 Results

The sociodemographic, medical condition, anthropometric, and body composition characteristics of the students are shown in Table 1. A total of 116 university students were included; 66 were female (56.9%), 50 were male (43.1%), and the mean age was 19.7 years (SD = 2). A total of 98 students (84.5%) did not present any diagnosed comorbidity (hypertension, diabetes mellitus, or hypercholesterolemia). According to the stratified BMI, 50 students (43.1%) had a normal weight, 45 (38.8%) had overweight, and 20 (17.2%) had obesity.

**Table 1:** Sociodemographic data, medical conditions, anthropometric data and body composition characteristics of the study sample.

Variables	Total (*n* = 116)	Male (*n* = 50)	Female (*n* = 66)	*p* value
Age (y), mean (SD)	19.7 (2)	19.7 (1.8)	19.8 (2.7)	0.879
Socioeconomic status, number (%)				
High	31 (26.7)	15 (12.9)	16 (13.7)	0.890^a^
Middle	85 (73.3)	35 (30.2)	50 (43.1)	**<0** **.** **001** ^a^
Low	0 (0)	0 (0)	0 (0)	0.890^a^
Work occupation, number (%)				
Full time	0 (0)	0 (0)	0 (0)	0.850^a^
Part time	27 (23.3)	15 (12.9)	12 (10.4)	0.762^a^
Not working	89 (76.7)	35 (30.2)	54 (46.5)	**<0** **.** **001** ^a^
Comorbidities (hypertension, diabetes mellitus, hypercholesterolemia), number (%)				
None	98 (84.5)	45 (38.8)	53 (45.7)	0.124^a^
Only one	17 (14.7)	4 (3.4)	13 (11.3)	**<0** **.** **001** ^a^
Two	1 (0.9)	1 (0.9)	0 (0)	0.340^a^
All the three	0 (0)	0 (0)	0 (0)	0.986^a^
Waist circumference (cm), mean (SD)	77.7 (8.7)	80.7 (7.5)	75.5 (8.9)	**0** **.** **005** *
Weight (kg), mean (SD)	61.9 (12.9)	69.8 (11.4)	56.4 (10.8)	**<0** **.** **001** *
Height (cm), mean (SD)	163 (0.9)	169 (0.7)	161 (0.5)	0.347*
BMI (kg/mt^2^), mean (SD)	23.7 (3.5)	24.2 (3.5)	23.3 (3.4)	0.092*
Stratified BMI, number (%)				
Underweight (<18.5)	1 (0.9)	0 (0)	1 (0.9)	0.513^a^
Normal (18.5–24.9)	50 (43.1)	22 (19)	28 (24.1)	0.064^a^
Overweight (25–29.9)	45 (38.9)	22 (19)	23 (19.9)	0.683^a^
Obesity (30–39.9)	20 (17.2)	6 (5.2)	14 (12)	**<0** **.** **001** ^a^
Extreme Obesity (>40)	0 (0)	0 (0)	0 (0)	0.972^a^
Fat mass index (kg/mt^2^), mean (SD)	6.6 (3.2)	5.3 (3.1)	7.7 (2.9)	0.069*
Lean mass index (kg/mt^2^), mean (SD)	16.9 (2.1)	18.8 (1.7)	15.6 (1.2)	0.211*
Total body water (%), mean (SD)	53.1 (6.9)	57.6 (5.6)	49.7 (5.9)	0.147*
Phase angle (°), mean (SD)	6.7 (0.7)	7.2 (0.8)	6.2 (0.6)	**0** **.** **067** *

BMI, Body mass index; SD, Standard deviation.

* Differences between males and females were obtained with *t*-tests; boldface indicates a *p* value < 0.05.

^a^ Differences between males and females were obtained with chi-square tests.

The lifestyle behavior characteristics of the students are shown in Table 2. Overall, the adherence to the 24-hour movement guidelines of the students upon admission to university was 8.6%. Conversely, 86 students (74.1%) did not meet any of the recommendations. There was a greater proportion of males who met all three 24-h movement recommendations (*p *= 0.025), physical activity recommendations (*p *= 0.035), sleep duration recommendations (*p* = 0.041), or sedentary behavior recommendations (*p *= 0.009) than females. A total of 48 students (41.4%) exhibited a high level of physical activity, 82 students (70.7%) needed changes in diet quality, 81 students (69.8%) experienced significant sleep disturbance (PSQI questionnaire > 5 points), and 22 students (18.9%) consumed harmful alcohol (AUDIT questionnaire > 8 points). Finally, according to the C4 smoking classification questionnaire, 68 students (58.6%) reported not smoking.

**Table 2:** Lifestyle behavior characteristics of the study sample.

Variables	Total (*n* = 116)	Males (*n* = 50)	Females (*n* = 66)	*p* value
GPAQ activity levels, number (%)				
High level	48 (41.4)	35 (30.2)	13 (11.2)	**0** **.** **005** ^a^
Moderate level	37 (31.9)	10 (8.6)	27 (23.7)	**<0** **.** **001** ^a^
Low level	31 (26.7)	5 (4.3)	26 (22.4)	**<0** **.** **001** ^a^
Total METs (week), mean (SD)	4,252 (4,640)	5,829 (4,920)	2,932 (4,050)	**<0** **.** **001** ^a^
Total PSQI questionnaire (points), mean (SD)	7.3 (3.1)	6.6 (2.9)	7.7 (3.3)	0.231*
Students with PSQI questionnaire (>5 points), number (%)	81 (69.8)	30 (25.9)	51 (43.9)	**<0** **.** **001** *
Physical activity recommendation, meeting (%)	12 (10.3)	8 (6.9)	4 (3.4)	**0** **.** **035** **^a^**
Sedentary behavior, meeting (%)	17 (14.7)	12 (10.4)	5 (4.3)	**0** **.** **009** **^a^**
Sleep duration, meeting (%)	18 (15.5)	11 (9.5)	7 (6)	**0** **.** **041** **^a^**
All three 24-hour movement guidelines, meeting (%)	10 (8.6)	7 (6)	3 (2.6)	**0** **.** **025** **^a^**
Dominant hand grip strength (kg), mean (SD)	29.8 (16.8)	40.9 (18.1)	22.1 (8.8)	**<0** **.** **001** *
Nondominant hand grip strength (kg), mean (SD)	27.1 (16.3)	38.3 (18.9)	19.2 (7.4)	**<0** **.** **001** *
Categories according HEI questionnaire, number (%)				
Healthy feeding	27 (23.3)	10 (8.6)	17 (14.7)	**<0** **.** **001** **^a^**
Need for change	82 (70.7)	35 (30.2)	47 (40.5)	**<0** **.** **001** **^a^**
Unhealthy feeding	7 (6)	5 (4.3)	2 (1.7)	**0** **.** **005** **^a^**
Total AUDIT questionnaire (points), mean (SD)	5.7 (6.4)	6.8 (6.9)	4.7 (5.9)	**<0** **.** **001** *
Students with AUDIT questionnaire (>8 points), number (%)	22 (18.9)	15 (12.9)	7 (6)	**<0** **.** **001** *
Smoking habits, number (%)				
No	68 (58.6)	30 (25.8)	38 (32.8)	0.082^a^
Moderate-risk smoker	13 (11.2)	5 (4.3)	8 (6.9)	0.073^a^
Occasional smoker	35 (30.2)	15 (12.9)	20 (17.2)	**0** **.** **025** **^a^**

METs, metabolic equivalents; GPAQ, Global Physical Activity Questionnaire; HEI, Healthy Eating Index; PSQI, Pittsburgh Sleep Quality Index; AUDIT, Alcohol Use Disorders Identification Test; SD, standard deviation.

* Differences between males and females were obtained with *t*-tests; boldface indicates a *p* value < 0.05.

^a^ Differences between males and females were obtained with chi-square tests.

Regarding the differences according to sex, compared with males, females reported a greater percentage of occasional smokers (*p *= 0.025) and a greater percentage of obesity (*p *< 0.001), a lower level of physical activity (*p *< 0.001), and a greater percentage of sleep disturbance (*p *< 0.001). Conversely, compared with females, males reported greater waist circumference (*p *= 0.005), weight (*p* < 0.001), handgrip strength (*p *< 0.001), levels of physical activity (*p *< 0.001), and percentages of harmful alcohol consumption (*p *< 0.001).

## 4 Discussion

This cross-sectional study aimed to describe sociodemographic characteristics, anthropometrics, body composition, and lifestyle behaviors of first-year students who entered physiotherapy school at the *Universidad de las Americas* in Quito, Ecuador, and compare them by sex. The main findings of our study were that less than one out of 10 university students met all three 24-hour movement guidelines, with a greater proportion of that meeting occurring in males than in females. Additionally, females reported a greater percentage of occasional smokers, more obese individuals, and more likely to sleep disturbance, in addition to lower levels of physical activity. Conversely, males exhibited increased physical activity and handgrip strength, in addition to increased harmful alcohol consumption.

However, temporal trends in the BMI of adolescents are highly variable across countries (43). There is a consensus that a critical time for the adoption of unhealthy lifestyle behaviors appears to be in late adolescence and early adulthood, a period that coincides with admission to university (3–7). Previous studies have shown that this period is associated with the adoption of unhealthy lifestyle behaviors (3, 5–7, 12–15). During this time, adolescents and young people adopt lifestyle habits that are likely to be sustained into later adulthood (5, 44). These unhealthy lifestyle behaviors may lead to weight and fat gain, which could have negative long-term health implications (5, 44–46).

To address obesity prevention, one approach involves identifying critical periods of weight gain throughout the lifespan, allowing for targeted interventions in prevention, promotion, and treatment during these pivotal times (6). Two systematic reviews have examined interventions targeting physical activity, diet, and weight-related behaviors specifically in university students (47, 48). One study suggested that university students represent ideal candidates for lifestyle interventions due to their academic environment rich in research expertise, multidisciplinary health professionals, and readily available facilities, potentially creating an optimal health-promoting setting. Additionally, being in a learning environment and at an age where health behaviors can significantly impact later life makes students receptive to interventions (47). Another study indicated that both face-to-face and electronic interventions demonstrated improvements in cognitive variables related to diet or physical activity and moderately influenced weight-related outcomes, albeit with less effectiveness in changing actual behaviors (48). Consistent with our findings, interventions targeting unhealthy lifestyle behaviors and weight gain in university students should consider distinct focuses for males and females.

While 98 students (84.5%) in the study did not report any diagnosed comorbidities such as hypertension, diabetes mellitus, or hypercholesterolemia, it's essential to recognize the strong connection between weight gain during adolescence and young adulthood and the increased likelihood of being overweight or obese in later adulthood (49). Over the medium or long term, a high body mass index (BMI) has been associated with an elevated risk of conditions such as hyperlipidemia, heart disease, hypertension, and stroke, among others (50). Therefore, it becomes crucial to identify, characterize, and assess the impact of specific determinant factors during critical periods that may contribute to obesity, subsequently affecting long-term overall health and well-being in this population.

In our sample, 50 students (43.1%) had a normal weight, 45 (38.9%) were overweight, and 20 (17.2%) had obesity according to their BMI status (28), with a greater proportion of females (12%) being obese than males (5.2%). Conversely, males had greater waist circumferences and body weights but not greater BMIs. Interestingly, few studies have reported similar rates of obesity/overweight among students upon admission to university (51, 52). However, additional quality longitudinal studies are needed to determine the determinants of changes in behavior across these life transitions, which are associated with deterioration of lifestyle behaviors and gains in adiposity as individuals enter adulthood (11).

Studying at university is often characterized by unhealthy changes in physical activity and dietary behavior with consequent weight gain (12, 48). Physical inactivity and an unhealthy diet are related behaviors that impact health and well-being and the maintenance of a healthy weight (47). Additionally, these behaviors underpin the risk of lifestyle-related noncommunicable conditions (53). Pathologies such as ischemic heart disease, stroke, type two diabetes, osteoporosis, various cancers, and depression are commonly associated with behavioral and biomedical health determinants, such as physical inactivity, poor dietary behaviors, and overweight/obesity (47). We found that only 48 students (41.4%) in the sample exhibited a high level of physical activity. In line with our findings, a meta-analysis showed consistent evidence of a decrease in physical activity and an increase in body weight across the transitions out of secondary school and into university (11). Moreover, one study reported a global physical inactivity prevalence of 41% (ranging from 22 to 81%) among students across universities in 23 low-, medium-, and high-income countries (18).

Adequate sleep duration plays a crucial role in promoting optimal physical health, immune function, mental health, and cognition (54). Unfortunately, attending university is often associated with insufficient sleep (55). Studies indicate that between 20% and 40% of university students (55, 56) do not meet the recommended sleep duration for their age group (7–9 h) (57). In our study, 81 students (69.8%) experienced significant sleep disturbance, as indicated by a PSQI score exceeding 5 points, with a higher percentage of females reporting sleep disturbance (*p* < 0.001). Interestingly, university students are recognized as a subset of the population particularly vulnerable to shortened sleep duration and/or sleep disruptions (56). Factors contributing to this include diminished parental support, increased stress from academic demands, engagement in unhealthy lifestyle behaviors, and irregularities in the sleep–wake cycle, leading to shortened and delayed sleep (58). It is noteworthy that sleep quality in this population has been identified as the strongest predictor of both physical and mental well-being (59).

Few published studies have evaluated adherence to the overall 24-hour movement guidelines among university students (60–63). Consistent with our findings, two studies reported similar rates of meeting recommendations among Canadian university students—9.9% and 8.4% (61, 62). Conversely, two other studies reported a greater proportion of Chinese (27%) and Portuguese (30.6%) university students meeting these recommendations (60, 63). One possible explanation for these differences could be related to cultural differences among countries. In this sense, a meta-analysis showed that the lowest prevalence of adherence to 24-hour movement recommendations was observed in Latin America (20). However, the lack of similar published studies of Latin American university students prevents us from comparing our findings.

Regarding handgrip strength, our results showed that males had greater handgrip strength than females did (*p* < 0.001). Several previous studies have analyzed handgrip strength in university students (64, 65). Consistent with our findings, one study of 276 physiotherapy students showed that males had significantly greater handgrip strength than females did (64). Another study also showed that males had greater handgrip strength than females did (65). This difference could be explained by several factors. One factor is that males have greater muscle mass than females, which could be related to a greater capacity for muscle strength. Another explanation could be associated with major skeletal muscle mass in males. Finally, males have a major tissue responsible for blood glucose disposal and an extensive reservoir of amino acids; thus, by having this greater reservoir of amino acids, the muscle contractile capacity to generate strength becomes greater (66, 67).

The prevailing understanding is that university students are susceptible to nutritional deficiencies due to unhealthy dietary habits (68). Upon entering university, individuals often adopt more westernized diets, characterized by low consumption of fresh fruits and vegetables, monounsaturated fatty acids, polyunsaturated fatty acids, and fish, coupled with increased intake of sugar, alcohol, and fast food (3, 5, 7, 12, 68, 69). Consistent with these observations, our study revealed that 82 students (70.7%) in the sample exhibited a need for improvements in diet quality and reported engaging in unhealthy eating habits. Within this population, potential contributing factors were identified, including changes in living arrangements, limited financial resources, lack of experience in meal planning, and an upsurge in the consumption of fast food and snacks (70).

Harmful alcohol consumption causes an increase in mortality and morbidity, including anxiety and depression symptoms, abuse of other drugs, cognitive deficits, cancer, heart disease, violence (including suicide), and road traffic accidents (71). In our study, 22 students (18.9%) consumed harmful alcohol (AUDIT questionnaire > 8 points), with a greater percentage of males (*p* < 0.001). In the literature, the results are controversial; one study reported a significant increase in alcohol intake in female students (72). Conversely, another study reported that males tended to consume more alcohol than females did (5). Consistent with our findings, another study of university students in Ecuador reported that males consumed alcohol significantly more than females did. However, the prevalence of harmful alcohol consumption above the cutoff score of eight in the AUDIT questionnaire was strikingly high. Approximately 50% of male students and 25% of female students reported harmful alcohol consumption (41).

Regarding smoking habits, 68 students (58.6%) reported not smoking, and females reported a greater percentage of occasional smokers (*p *= 0.025). In contrast with these findings, a study performed in Latin American smokers showed a tendency toward greater consumption in terms of frequency and intensity in males, and females reported lighter and less intense consumption, but that was no less risky for their health (42). In this sense, one study showed that the main determinants of regular smoking behavior in first-year university students were male sex, low academic performance, having smoked friends, high level of income, and a mother with a high education level (73).

This study has several limitations. First, as this was a cross-sectional (data from all students were assessed at a single point in time) and observational study, it did not include a comparison group of young adults who did not attend university but who may also have significant environmental or lifestyle changes; therefore, causality cannot be established. Second, there is a lack of information on changes in anthropometrics, body composition, and lifestyle variables during medium- or long-term follow-up. Third, self-report questionnaires were used for assessment and are prone to recall and desirability bias. Fourth, our study was performed only in students who entered a physiotherapy school; therefore, our findings cannot be generalized to all students who entered university. Finally, these limitations should be considered when attempting to extrapolate our findings to all university students. Conversely, several strengths can be highlighted. For instance, only a few previous studies have analyzed adherence to 24-hour movement guidelines, anthropometrics, body composition, and lifestyle behavior upon admission to university among students in Latin American countries, and none have analyzed adherence to 24-hour movement guidelines in Ecuador. Although the sample size is not representative of the total population of Ecuadorian university students, this is a pilot study that will serve as a basis for subsequent studies in this population. Furthermore, analyzing the results of these variables by sex could be useful for developing more effective sex-specific interventions to improve lifestyle behaviors during this critical period.

## 5 Conclusions

This study serves as a starting point for future studies that analyze changes in anthropometry, body composition, and lifestyle behaviors (including adherence to 24-hour movement guidelines) upon admission to university in Latin American students. Our results showed that overall adherence to these guidelines is low among university students. Additionally, a high percentage of students reported unhealthy lifestyle behaviors, with differences according to sex. Identifying critical periods for the adoption of unhealthy movement and lifestyle behaviors and the factors that influence them may lead to the development of effective prevention programs. Therefore, there is an urgent need for the implementation of movement and lifestyle interventions to improve the health of this group, which represents a significant proportion of our population. However, additional research is needed in Latin American universities, especially in Ecuador.

## Data Availability

The raw data supporting the conclusions of this article will be made available by the authors, without undue reservation.
